# Xenobiotic-Induced Aggravation of Metabolic-Associated Fatty Liver Disease

**DOI:** 10.3390/ijms23031062

**Published:** 2022-01-19

**Authors:** Julie Massart, Karima Begriche, Anne Corlu, Bernard Fromenty

**Affiliations:** Institut NUMECAN (Nutrition Metabolisms and Cancer), UMR_A 1341, UMR_S 1241, INSERM, University Rennes, INRAE, F-35000 Rennes, France; karima.begriche@univ-rennes1.fr (K.B.); anne.corlu@inserm.fr (A.C.); bernard.fromenty@inserm.fr (B.F.)

**Keywords:** obesity, fatty liver, NASH, drugs, environmental contaminants, endocrine disruptors, ethanol

## Abstract

Metabolic-associated fatty liver disease (MAFLD), which is often linked to obesity, encompasses a large spectrum of hepatic lesions, including simple fatty liver, steatohepatitis, cirrhosis and hepatocellular carcinoma. Besides nutritional and genetic factors, different xenobiotics such as pharmaceuticals and environmental toxicants are suspected to aggravate MAFLD in obese individuals. More specifically, pre-existing fatty liver or steatohepatitis may worsen, or fatty liver may progress faster to steatohepatitis in treated patients, or exposed individuals. The mechanisms whereby xenobiotics can aggravate MAFLD are still poorly understood and are currently under deep investigations. Nevertheless, previous studies pointed to the role of different metabolic pathways and cellular events such as activation of de novo lipogenesis and mitochondrial dysfunction, mostly associated with reactive oxygen species overproduction. This review presents the available data gathered with some prototypic compounds with a focus on corticosteroids and rosiglitazone for pharmaceuticals as well as bisphenol A and perfluorooctanoic acid for endocrine disruptors. Although not typically considered as a xenobiotic, ethanol is also discussed because its abuse has dire consequences on obese liver.

## 1. Introduction

Obesity and associated diseases meet epidemic proportions in numerous countries. In particular, the hepatic tissue can rapidly suffer from the accumulation of lipids, thus leading to fatty liver and other liver lesions. The whole spectrum of hepatic lesions linked to obesity is called metabolic (dysfunction)-associated fatty liver disease (MAFLD), which is now preferred to non-alcoholic fatty liver disease (NAFLD) [1]. Obesity is associated with insulin resistance and type 2 diabetes mellitus (T2DM), hypertension, dyslipidemia and osteoarthritic disorders. All these conditions may need the prescription of different types of pharmaceuticals, such as antidiabetic and antihypertensive drugs, lipid-lowering agents or non-steroidal anti-inflammatory drugs. Hence, obese individuals are more likely to have long-term polypharmacy [2,3]. In addition, obese people, as lean ones, can be exposed, voluntarily or accidentally, to many other xenobiotics, such as recreational drugs, plant and mushroom toxins, or industrial chemicals.

**Figure 1:**
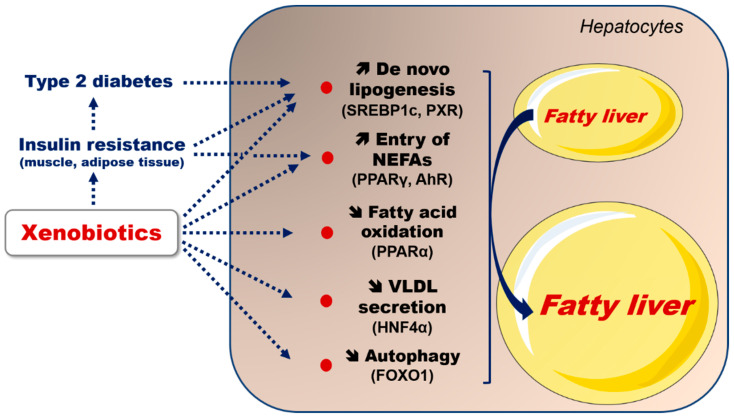
Mechanisms whereby xenobiotics can aggravate obesity-related fatty liver. Xenobiotics can exacerbate pre-existent lipid accumulation in hepatocytes by direct mechanisms such as stimulation of de novo lipogenesis (DNL), increased non-esterified fatty acid (NEFA) uptake, reduction in fatty acid oxidation and impairment of very low-density lipoprotein (VLDL) secretion. Stimulation of DNL results from the activation of different lipogenic nuclear receptors such as PPARγ and PXR. Alternatively, increased DNL is secondary to insulin resistance, for instance, at the level of skeletal muscle and adipose tissue. Insulin resistance leads to hyperinsulinemia, which activates SREBP1c in hepatocytes. Insulin resistance in white adipose tissue also favors triacylglycerol lipolysis, thus leading to the unrestrained release in blood of NEFAs freely entering the liver via FAT/CD36 or other fatty acid transporters. If insulin resistance progresses to type 2 diabetes, hyperglycemia increases hepatic DNL via the activation of ChREBP. Reduced fatty acid oxidation can be attributed to different mechanisms, including reduced PPARα activity, or direct impairment of mitochondrial enzymes. Reduced VLDL secretion can be secondary to endoplasmic reticulum stress. Finally, impairment of autophagy favors lipid accumulation by reducing the clearance of excessive lipid droplets. Some connection arrows are not mentioned for the sake of clarity. Further information is provided in the text.

**Figure 2:**
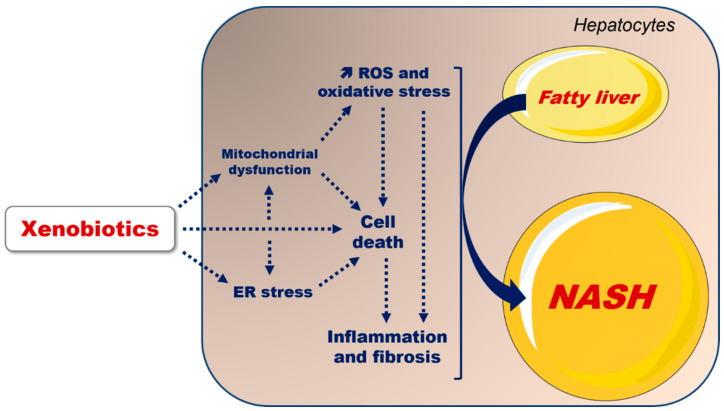
Mechanisms whereby xenobiotics can favor the progression of obesity-related fatty liver to NASH, characterized by necroinflammation, hepatocyte ballooning, apoptosis and fibrosis, in addition to steatosis. Mitochondrial dysfunction, in particular at the level of the respiratory chain, leads to reactive oxygen species (ROS) overproduction, which, in turn, induces oxidative stress. Mitochondrial dysfunction, ROS overproduction and oxidative stress trigger cell death by necrosis or apoptosis. Cell death can also be induced by different cytokines such as TNF-αand Fas ligand. ROS overproduction and hepatocyte cell death favor inflammation and fibrosis through the activation of Kupffer cells and stellate cells, respectively. Finally, endoplasmic reticulum (ER) stress can also lead to cell death and oxidative stress (not shown). Note that there is an interplay between mitochondrial dysfunction and ER stress. Some connection arrows are not mentioned for the sake of clarity. Further information is provided in the text.

An increasing number of investigations of obese individuals and rodent models indicate or strongly suggest that some xenobiotics can further increase hepatic lipid levels (Figure 1), or accelerate the transition from fatty liver to steatohepatitis or cirrhosis (Figure 2). Unfortunately, the mechanisms of these deleterious effects are not well understood, in particular because many investigations are only observational. Nonetheless, some experimental studies allow different hypotheses to be proposed in order to explain the exacerbation of fatty liver, or the faster occurrence of steatohepatitis. In this review, we first recall the main features of MAFLD regarding its physiopathology. Next, we review some xenobiotics which can worsen MAFLD, including pharmaceuticals and industrial chemicals. Finally, we discuss ethanol, although this molecule is not typically considered as a xenobiotic. Indeed, there is now ample evidence that ethanol abuse has dire consequences on the obese liver.

## 2. Main Features of MAFLD

### 2.1. Clinical Features, Liver Pathology and Blood Chemistry

While the classical term NAFLD refers to fatty liver disease in absence of excessive alcohol consumption, which is quite restrictive, MAFLD more appropriately places this disease in the broader context of metabolic dysfunction [1]. In fact, hepatologists wished to follow the example of other medical specialties such as cardiology, diabetology and oncology, which have successfully distanced the disease from underlying obesity, smoking, alcohol overconsumption and drug abuse [1]. The whole spectrum of hepatic lesions in MAFLD is the same as NAFLD and includes fatty liver (also referred to as hepatic steatosis), steatohepatitis (NASH), cirrhosis and hepatocellular carcinoma [4]. Most obese individuals present simple fatty liver but this lesion can progress, in the long term, to NASH in 10–20% of subjects. In obesity-associated fatty liver, lipids accumulate mainly as macrovacuolar steatosis, but microvesicular steatosis is also observed and seems to be linked to more severe forms of MAFLD [5], possibly due to severe impairment of mitochondrial fatty acid oxidation (FAO) [6,7]. NASH is characterized by necroinflammation, hepatocyte ballooning and some degree of fibrosis, in addition to steatosis. Apoptotic hepatocytes, megamitochondria, Mallory-Denk bodies and iron accumulation can also be observed [5,8]. At these stages of the disease, patients are often asymptomatic but some of them present non-specific symptoms, such as fatigue, right upper quadrant discomfort and hepatomegaly [9]. NASH can then progress, in some individuals, to advanced cirrhosis, which is associated with bridging fibrosis and nodularity as well as canalicular cholestasis [5]. When cirrhosis occurs, ascites can cause abdominal distention, nausea and vomiting, dyspnea and lower-extremity edema. Late complications of cirrhosis include variceal hemorrhages, hepatic encephalopathy, spontaneous bacterial peritonitis and sepsis. Accordingly, the mortality of patients with decompensated cirrhosis is high.

Blood chemistry in MAFLD can include mild elevation of alanine aminotransferase (ALT) and aspartate aminotransferase (AST) activities, thus reflecting the presence of slight hepatic cytolysis, but a significant number of patients can have normal aminotransferase levels [10,11]. When the disease progresses to cirrhosis, hyperbilirubinemia, hyperammonemia, hypoalbuminemia and abnormal prothrombin time can be observed [9,10].

### 2.2. Physiopathology of MAFLD

The physiopathology of MAFLD is complex and is still poorly understood. Here, we briefly review the main features of MAFLD pathogenesis, although several recent reviews are available for more details on this subject [8,12,13,14,15,16,17].

Insulin resistance in white adipose tissue (WAT) and skeletal muscle plays a major role in the pathogenesis of obesity-related fatty liver [12,14,15,17,18]. Insulin resistance in WAT favors triacylglycerol lipolysis, thus leading to a massive release, into the circulation, of glycerol and non-esterified fatty acids (NEFAs), which enter the liver in a concentration-dependent manner via transporters such as the fatty acid translocase (FAT/CD36) and different fatty acid transport proteins (FATPs). Furthermore, fatty acids are synthesized actively in the liver because insulin resistance-associated hyperinsulinemia favors de novo lipogenesis (DNL; i.e., the synthesis of fatty acids from carbohydrates) via the activation of sterol regulatory element-binding protein 1c (SREBP1c). Insulin resistance in skeletal muscle impairs glucose uptake and glycogen synthesis, thus favoring glucose utilization for hepatic DNL. In addition, hepatic DNL can be activated by carbohydrate response element binding protein (ChREBP) when hyperglycemia occurs [13,19]. Finally, whereas mitochondrial FAO is not reduced in the early stage of MAFLD, impaired autophagy can favor lipid droplets accumulation [16,20]. Indeed, a defect in autophagy reduces the clearance of lipid droplets, further promoting steatosis development [20].

The pathogenesis of fatty liver progression to NASH seems to involve multiple hits and targets as extensively discussed in previous reviews [8,12,14,15,17,21,22,23] (Figure 2). Briefly, evidence points to a major role of mitochondrial dysfunction, overproduction of reactive oxygen species (ROS), reduced ROS detoxification and endoplasmic reticulum (ER) stress. Of note, ER stress seems to also play an important role in hepatic lipid accumulation via different mechanisms, including impairment of very low-density lipoprotein (VLDL) secretion [21,24]. ROS overproduction during MAFLD might mainly occur within mitochondria, in particular at the level of different complexes of the mitochondrial respiratory chain (MRC) and some enzymes of the FAO pathway [23,25]. However, another source of ROS in MAFLD seems to be cytochrome P450 2E1 (CYP2E1), as discussed below. Other important factors involved in MAFLD progression include increased production of pro-inflammatory and profibrogenic cytokines by parenchymal and non-parenchymal liver cells. Lastly, besides the exposure to different xenobiotics as discussed in this review, genetic polymorphisms in different genes (e.g., *PNPLA3* and *TM6SF2*) could be involved in fatty liver progression to NASH in a subset of obese subjects.

Metabolic “flexibility” or “adaptation” is an important feature of MAFLD, at least in the early stages of the disease. For instance, higher hepatic secretion of VLDL is observed in patients with obesity-related fatty liver [13,23,26]. Increased mitochondrial FAO and tricarboxylic acid cycle activity also occurs in simple fatty liver [15,23,27]. However, these metabolic adaptions are still not sufficient to avoid hepatic lipid accumulation. Furthermore, mitochondrial flexibility appears to play a major role in oxidative stress and inflammatory processes [23,27]. Notably, mitochondrial adaptations are apparently lost during NASH, which might further favor ROS overproduction [23,28]. The occurrence of mitochondrial “inflexibility” could involve a progressive reduction in MRC activity and a loss of peroxisome proliferator-activated receptor alpha (PPARα) expression and activity [23,28,29].

### 2.3. MAFLD and Changes in Xenobiotic Metabolism

Another metabolic feature of MAFLD and obesity is the altered hepatic expression and activity of numerous xenobiotic metabolizing enzymes (XMEs), extensively discussed in several previous reviews [30,31,32,33,34]. XME activity is also altered in the intestine and kidneys of obese individuals, further impacting drug pharmacokinetics [33]. In addition to metabolism, the alteration in drug absorption, distribution and elimination is observed in obesity due to altered gastric emptying and gut permeability, higher cardiac output and increased volume of distribution and glomerular filtration [33,35,36]. Although the impact of obesity and associated diseases (e.g., MAFLD and diabetes) on drug pharmacokinetics have been explored in many studies [32,33,34,35,37], much less is known regarding other xenobiotics such as industrial chemicals. Nevertheless, it is conceivable that several molecules present altered pharmacokinetics in obese people, which might change their toxicological profile.

MAFLD is associated with changes in the activity of some CYPs. For instance, human MALFD is frequently associated with reduced CYP3A4 activity and increased CYP2E1 activity [32,33,34,35,37,38]. Thus, the metabolism of drugs, such as troglitazone metabolized by CYP3A4 or acetaminophen by CYP2E1, as well as their hepatotoxicity profile, may be altered [39,40]. The mechanism of reduced CYP3A4 activity in MAFLD is still poorly understood, although two hypotheses can be put forward. Inflammation might be involved, since CYP3A4 expression and activity were found to be markedly reduced by different pro-inflammatory cytokines, such as IL1β and IL6 [41,42]. Whether decreased CYP3A4 activity plays a role in MAFLD progression remains unclear. Some investigations also pointed to a role of the fibroblast growth factor 21-pregnane X receptor (PXR) pathway, but others failed to demonstrate any involvement of PXR [38]. Likewise, the mechanism of MAFLD-associated CYP2E1 induction is still unclear. Previous investigations pointed to a role of some fatty acids such as stearic acid [43,44]. In addition, insulin resistance-associated hyperinsulinemia and increased glycemia might also play a role in some obese individuals [45]. Lastly, MAFLD is associated with changes in the activity of XMEs other than CYPs, such as uridine diphosphate glucuronosyltransferases (UGTs), which can be associated with higher glucuronide formation, at least with some drugs, including lorazepam, oxazepam and acetaminophen [35,38].

Increased CYP2E1 activity in MAFLD might explain why some drugs such as acetaminophen and halothane induce more severe hepatic liver injury in obese patients, as discussed in previous reviews [40,46,47]. Besides its effect on drug-induced liver injury, higher CYP2E1 activity seems to play a significant role in the progression of fatty liver to NASH [43,48,49,50]. Indeed, the induction of CYP2E1 could favor oxidative stress, because this enzyme produces significant amounts of superoxide anion during its catalytic cycle [43,48]. Noteworthy, CYP2E1 is located within the ER and the mitochondria, thus producing ROS and inducing oxidative stress in both compartments [51,52]. Thus, endogenous or exogenous compounds able to enhance hepatic CYP2E1 activity are expected to promote the transition from fatty liver to NASH. Finally, some investigations suggested that CYP2E1 induction exacerbates hepatic lipid accumulation, possibly via an ROS-mediated reduction in PPARα activity [47,53].

## 3. Xenobiotics Able to Aggravate MAFLD

In this section, we discuss selected drugs and environmental toxicants which could worsen MAFLD through different mechanisms (Figure 1). The molecules described in this section were chosen based on sufficient supporting clinical investigations and experimental data. However, readers are invited to peruse previous reviews in order to know more about xenobiotics that are not discussed in detail below. This concerns drugs such as irinotecan, methotrexate, nucleoside reverse transcriptase inhibitors, phenobarbital and tamoxifen (Table 1) [40,47]. Although not typically considered as a xenobiotic, we also discuss ethanol because there are now numerous studies reporting that its abuse has harmful consequences on the liver of obese alcoholics.

### 3.1. Drugs

#### 3.1.1. Corticosteroids

Corticosteroids (also referred to as glucocorticoids) are potent anti-inflammatory molecules used to treat lupus, rheumatoid arthritis, asthma, Crohn disease and different types of allergies. Inflammatory liver diseases such as alcoholic hepatitis are routinely treated with corticosteroids [54], while their use is not recommended for MAFLD [55]. Corticosteroids include natural hormones, such as cortisol and corticosterone, and synthetic agents, including dexamethasone, prednisone and triamcinolone. Corticosteroids can induce, in some patients, different types of liver lesions, such as hepatic cytolysis, steatosis, steatohepatitis and benign liver tumors [40,56]. Moreover, these drugs can favor abdominal obesity, hyperlipidemia, insulin resistance and T2DM, even after topical administration [47,57,58].

Despite numerous studies on corticosteroid-induced effects on lipid metabolism [59,60], the mechanisms whereby these molecules can induce hepatic steatosis only in a subset of patients are poorly understood and sometimes controversial [61]. For instance, some studies reported that corticosteroids directly induce triglyceride accumulation in cultured hepatocytes, in particular through increased uptake and synthesis of fatty acids [62,63]. In contrast, other investigations failed to report dexamethasone-induced triglyceride accumulation in cultured hepatocytes, thus leading to the proposal that corticosteroid-induced steatosis in vivo requires some systemic factors such as NEFAs or periostin [61,64]. Hence, corticosteroids may favor lipid accumulation in the liver through different mechanisms depending on substrate availability, systemic factors or binding partners [55,62,63]. Indeed, the glucocorticoid receptor (GR) interacts with numerous hepatocyte-expressed transcription factors playing a major role in lipid metabolism, such as PPARα, Forkhead box protein O1 (FOXO1), Hepatocyte Nuclear Factor 4α (HNF4α) and Liver X Receptor (LXR) [60]. Obviously, further investigations are required to decipher the molecular mechanisms involved in corticosteroid-induced steatosis.

Given the extensive effects on lipid homeostasis, corticosteroids may worsen pre-existing metabolic disturbances and MAFLD (Table 1) [40,47]. Although not reported in obese individuals yet, long-term treatment with dexamethasone worsened hepatic triglyceride accumulation in diet-induced obese mice [65,66]. Importantly, liver triglycerides were not increased in mice fed a standard diet, thus indicating a synergistic effect of high-fat diet (HFD) and dexamethasone on steatosis [65,66]. However, in rats, an additive effect between corticosterone and HFD on hepatic lipid deposition was found, as corticosteroid-treated animals fed a standard diet developed hepatic steatosis [67,68]. In one of these studies, plasma bilirubin and ALT levels, and liver collagen content were significantly increased in HFD rats treated with corticosterone compared to untreated HFD rats [67]. Accordingly, these results suggest that corticosteroids might not only exacerbate liver lipid accumulation but might also favor the transition from simple fatty liver to NASH.

The mechanisms whereby corticosteroids worsen obesity-related fatty liver in rodents remain largely unknown. A first suspected mechanism is insulin resistance, associated with increased [67,68] or decreased [66] body fat mass. In the latter study, the reduction in adiposity was correlated with an upregulation of the lipolytic enzyme adipose triglyceride lipase (ATGL), although increased energy expenditure might also be involved [66]. However, these investigations did not unveil how corticosteroids and obesity could synergistically activate ATGL expression [66]. In addition to insulin resistance and increased flux of NEFAs to the liver, indirect evidence also suggests that corticosteroids and obesity might favor hepatic steatosis via enhanced circulating levels of periostin, an extracellular matrix protein expressed in most tissues, including WAT [61] and liver [69]. Dexamethasone-induced steatosis in mice was almost fully prevented by the administration of a periostin-neutralizing antibody or in periostin-knockout mice [61]. In this study, the prevention of dexamethasone-induced steatosis was associated with a restoration of hepatic PPARα expression [61]. Besides, obese patients with MAFLD present high circulating levels of periostin [69,70,71]. Moreover, increased hepatic expression of periostin associated with MAFLD negatively regulates PPARα expression via a mechanism dependent on c-Jun N-terminal kinase (JNK) [69]. Nevertheless, it remains to be determined whether corticosteroids and obesity exert synergistic effects on periostin-induced PPARα downregulation and alteration in lipid metabolism in the liver.

#### 3.1.2. Thiazolidinediones

Thiazolidinediones are synthetic antidiabetic agents whose beneficial effect on insulin sensitivity is mediated via PPARγ activation. This drug class includes different molecules, such as rosiglitazone, troglitazone and pioglitazone.

Rosiglitazone is an analogue of troglitazone, the first derivative of the thiazolidinedione family withdrawn from the market in 2000. Indeed, troglitazone induced several cases of fatal acute liver injury with massive hepatic cytolysis, cholestasis and microvesicular steatosis [40,72,73]. Rosiglitazone is much safer than troglitazone, although several cases of hepatic cytolysis and cholestasis, sometimes severe, were reported in treated individuals [74]. In contrast, to our knowledge, rosiglitazone has not been reported to induce microvesicular steatosis in patients. Noteworthy, rosiglitazone therapy is associated with body weight gain, peripheral edema and heart failure, so that this antidiabetic is prescribed as a second-line agent for T2DM. Rosiglitazone-induced cardiovascular side effects led different countries to withdraw this drug from the market. The second thiazolidinedione derivative currently approved by the Food and Drug Administration (FDA) is pioglitazone.

Both rosiglitazone and pioglitazone have been tested in the treatment of NASH [40]. Although pioglitazone therapy showed consistent beneficial effects on NASH progression, rosiglitazone use was overall less effective than pioglitazone or found to be without significant benefit in some investigations [75,76]. In addition, rosiglitazone-induced worsening of steatosis, necroinflammation and perisinusoidal fibrosis were observed in some patients (Table 1) [47,77]. In keeping with these observations, patients treated for 12 months with rosiglitazone showed increased hepatic mRNA expression of Toll-like receptor 4 (*TLR4*), interleukin-8 (*IL8*) and C-C motif chemokine ligand 2 (*CCL2*, also known as MCP-1), thus suggesting a pro-inflammatory state [78].

Several investigations performed on different murine models of obesity and T2DM reported that rosiglitazone worsened liver triglyceride accumulation and hepatic steatosis [79,80,81,82,83,84,85,86]. However, only a few studies compared rosiglitazone effects between lean and obese mice. In such investigations, rosiglitazone did not induce steatosis in lean animals, whereas it exacerbated hepatic fat accumulation in obese mice, demonstrating a synergistic effect of rosiglitazone and obesity on fatty liver [82,86]. In two aforementioned studies, rosiglitazone-induced aggravation of fatty liver was associated with increased circulating ALT activity, thus suggesting that necroinflammation could be also exacerbated [80,83]. In contrast to these studies, others showed that rosiglitazone alleviates hepatic steatosis in obese rodents [87,88,89]. The exact reasons of these discrepancies are still unknown but might be explained by several experimental parameters, such as differences in rodent models of obesity and protocols of rosiglitazone treatment, including duration and dose.

Rosiglitazone-induced worsening of hepatic triglyceride accumulation in obese mice was associated, in most investigations, with an improvement of insulin resistance, in keeping with its pharmacological action [80,81,82,84,85]. Yet, effects on body weight were not consistent, being either increased [80,81,82], unchanged [84] or reduced [85]. Unfortunately, these studies did not determine whether body fat mass was affected. Hence, further investigations are warranted to determine the relationship between the worsening of hepatic steatosis and adiposity.

Several studies attempted to decipher the mechanisms underlying the synergistic effect of obesity and rosiglitazone on fatty liver. Some investigations suggested an exacerbation of hepatic DNL [80], which might have been favored by the high basal expression of PPARγ in obese liver [83,86]. This hypothesis was reinforced by in vitro investigations of mouse hepatocytes overexpressing PPARγ2 [90]. Indeed, although troglitazone was used as PPARγ agonist in this study, intracellular lipid accumulation induced by PPARγ2 overexpression was further enhanced by this thiazolidinedione derivative [90]. In addition to exacerbated DNL, rosiglitazone could also favor uptake of NEFAs by the fatty liver, presumably via an increased expression of *FAT/CD36* [81]. 

Greater hepatotoxicity of rosiglitazone in some obese patients might also be secondary to an alteration in its pharmacokinetics, which might favor higher plasma concentrations and PPARγ overactivation in the liver. In keeping with this hypothesis, the plasma half-life of rosiglitazone was nearly tripled in a mouse model of diet-induced obesity [91]. Rosiglitazone mainly undergoes CYP2C8-mediated *p*-hydroxylation and N-demethylation, followed by sulfate and glucuronide conjugation [92], metabolic pathways that might be affected in obesity. A previous study suggested reduced CYP2C8 activity in obese individuals [93], but this was not confirmed by other investigators [94]. Hence, further investigations are required for a better understanding of rosiglitazone pharmacokinetics in obesity.

The mechanisms involved in rosiglitazone-induced hepatic necroinflammation in some subjects with MAFLD remain unknown. Rosiglitazone might exacerbate MAFLD-associated mitochondrial dysfunction, which, in turn, might cause overproduction of ROS and proinflammatory cytokines [23,27,83]. In keeping with this assumption, relatively low concentrations (25 and 50 µM) of rosiglitazone were shown to inhibit the activity of MRC complexes I–IV in differentiated HepaRG cells, which was associated with ROS overproduction [95]. More recent investigations of MDA-MB-231 cells confirmed that rosiglitazone could induce mitochondrial dysfunction, but ROS were not assessed in this study [96].

In contrast to rosiglitazone, pioglitazone showed consistent beneficial effects on NASH progression in patients [75,76], as previously mentioned. Nevertheless, investigations of obese mice showed that pioglitazone worsened fatty liver [97,98]. In one of these studies, pioglitazone increased the mRNA expression of several genes involved in lipid synthesis, such as fatty acid synthase (*Fasn*), stearoyl-CoA desaturase (*Scd1*) and enzymes involves in fatty acid elongation (*Elovl3*, *-5* and *-7*) [98]. However, pioglitazone concomitantly decreased the expression of different genes involved in the inflammatory response [98]. Interestingly, pioglitazone but not rosiglitazone might elicit anti-inflammatory effects via PPARα activation [99]. Hence, the anti-inflammatory property of pioglitazone might explain why this thiazolidinedione derivative presents more favorable effects on NASH progression than rosiglitazone. In addition, it is noteworthy that pioglitazone is a weaker human PPARγ activator than rosiglitazone [100,101], which could explain the differential steatogenic effect on patients.

#### 3.1.3. Other Drugs

MAFLD may be worsened, in obese patients, by other pharmaceuticals, such as irinotecan, methotrexate and tamoxifen (Table 1), which have been extensively discussed in previous reviews [40,47,102,103]. However, despite being observed clinically, the mechanisms leading to aggravation of MAFLD are poorly characterized. Pentoxifylline, a drug tested in NASH for its anti-TNF-γ activity, is suspected to aggravate fatty liver, inflammation and fibrosis in few patients (Table 1), although this methylxanthine derivative showed some beneficial effects in most patients [104,105]. A 3-week treatment with pentoxifylline aggravated fatty liver in obese diabetic ob/ob mice and this was associated with hepatic ChREBP overactivation, possibly as a consequence of enhanced intestinal glucose absorption and increased postprandial glycemia [105]. Notably, pentoxifylline did not induce steatosis in wild-type (i.e., lean) mice [105]. Hence, it is possible that long-term treatment with pentoxifylline could exacerbate fatty liver only in a subset of patients with severe pre-existing hyperglycemia. Other drugs, such as phenobarbital [106] and tetracycline [107] (Table 1), exacerbated fatty liver in obese rodents, yet clinical investigations with these drugs are lacking. Stavudine and didanosine are suspected to aggravate fatty liver in some patients, potentially through mitochondrial dysfunction [40]. However, experimental studies are required to determine the exact mechanisms.

### 3.2. Environmental Toxicants

#### 3.2.1. Bisphenol A

Bisphenol A (BPA) was identified as an estrogenic derivative in the mid-1930s, but this synthetic chemical was never developed as a drug [108]. Instead, BPA has been extensively used as a plasticizer incorporated in numerous consumer goods, such as food storage containers, bottles and CDs. Human exposure to BPA is suspected to favor obesity and related metabolic disorders, such as insulin resistance, T2DM and MAFLD [109,110,111]. Many experimental data in rodents confirmed that metabolic disturbances occur after exposure to BPA in adulthood or during the perinatal period [112,113]. Hence, BPA is now deemed to be a prototypical endocrine disruptor whose use in food containers and thermal paper has been banned in different countries [110].

The mechanisms of BPA-induced metabolic disturbances are complex and have been extensively discussed in recent reviews [110,111,113,114]. Briefly, many of the metabolic and endocrine alterations induced by BPA could be mediated via the activation of several nuclear receptors, such as estrogen receptor alpha (ERα), ERβ estrogen related receptor γ (ERRγ) and PXR. BPA could also activate PPARγ and CCAAT/enhancer binding proteins (C/EBPs), although it is still debated whether these transcription factors play a role in BPA’s mode of action [113,114]. Lastly, rodent studies suggested a role for SREBP activation in the liver [115,116], but whether such activation is direct or mediated by hyperinsulinemia remains to be determined.

The mechanisms whereby BPA can induce steatosis are still poorly understood. BPA-induced hepatic lipid accumulation is likely secondary to obesity and insulin resistance [111,117], while a recent study suggested a role for gut microbiota dysbiosis [118]. BPA could also have direct effects on hepatocytes. Indeed, several in vitro studies showed that BPA induced lipid accretion in the human hepatic cell lines HepG2, HepaRG and HHL-5 [119,120,121,122,123,124,125,126]. This effect was observed for BPA concentrations in the nanomolar range in four independent studies [119,120,123,125], with a non-monotonic profile observed in three of them [119,123,125]. Interestingly, in HepG2 cells, BPA increases SREBP1 expression through the downregulation of miR-192, thus leading to triglyceride accumulation [124]. Alternatively, BPA indirectly activates the endocannabinoid receptor CB1 to induce SREBP1 [122]. Of note, BPA-induced intracellular cholesterol accumulation involves SREBP2 activation [125]. A previous study suggested a role for mitochondrial dysfunction in BPA-induced neutral lipid accumulation, but mitochondrial FAO has not been assessed [120]. It has also been proposed that PXR may play a role [121], but this hypothesis could not be confirmed [123]. The latter study also provided evidence that ERRγ is not involved in BPA-induced steatosis [123].

Only a few studies compared the metabolic and hepatic effects of BPA between lean and obese mice. One study found a synergistic effect of BPA and diet-induced obesity regarding hepatic triglyceride content and collagen deposition [127]. Although this study did not provide mechanistic explanations for these effects, other studies showed a synergistic effect of BPA and HFD on glucose intolerance and, possibly, insulin resistance [128,129]. Maternal exposure to BPA led to increased hepatic lipid accumulation in rat male offspring, which was further exacerbated by HFD after weaning with a higher ratio of microvesicular/macrovacuolar steatosis [130]. Further investigations in this study suggested impaired mitochondrial FAO, especially at the level of carnitine palmitoyltransferase 1 (CPT1), a major enzyme involved in the mitochondrial entry of long-chain fatty acids [130].

The synergistic effects of BPA and HFD on fatty liver, glucose intolerance and possibly insulin resistance might be, at least in part, secondary to an impairment of BPA biotransformation resulting in higher BPA bioavailability. A previous study in humans and mice disclosed a strong reduction in BPA sulfonation in MAFLD [131]. However, reduced BPA sulfonation in MAFLD might have little consequence on BPA bioavailability, because BPA-sulfate is a minor metabolite of BPA in humans and rodents, in contrast to BPA-glucuronide [131,132]. Hence, future investigations are required to determine whether MAFLD is also associated with the impairment of BPA glucuronidation.

#### 3.2.2. Perfluorooctanoic Acid

Perfluorooctanoic acid (PFOA) is one of the most common poly- and perfluoroalkyl substances (PFAS) used for industrial and commercial applications. PFOA is a surfactant with water-repellent properties which can be found in many consumer goods, such as non-stick pans, weatherproof garments, floor waxes, food containers and cosmetics. Most uses of this chemical are banned or are progressively being suppressed in many countries because of suspected toxicity in animals and humans [133]. Indeed, the exposure to high levels of PFOA among residents living near the DuPont Teflon-manufacturing plant in Parkersburg, West Virginia, is linked to several types of cancers, especially testicular and kidney malignancies [134,135]. In addition, workers of this chemical plant presented a higher risk of mortality for several diseases, such as chronic renal diseases and diabetes mellitus [136]. Interestingly, longitudinal cohort studies suggested that environmental PFOA exposure in the general population might be associated with increased risk of T2DM [137,138]. Environmental PFOA exposure might also be associated with excess adiposity, in particular in the setting of early-life exposure [139,140], although other studies did not confirm these findings [141,142]. In contrast, the environmental PFOA exposure of the general population is consistently associated with hypercholesterolemia [135,141,143]. However, very high PFOA exposure seems to reduce total blood cholesterol levels, possibly via PPARα activation [144].

Regarding the liver, PFOA exposure might be associated with elevated ALT activity [135,142,145]. In some epidemiological studies, higher circulating ALT activity is more pronounced in obese subjects compared to nonobese ones, suggesting a synergistic effect of PFOA exposure and obesity on hepatic cytolysis [146,147]. In contrast, other investigations did not find an association between PFOA and elevated ALT activity [144]. Actually, except for hepatic cytolysis, the current available epidemiological data do not support a link between PFOA exposure and liver diseases, including MAFLD [133,135,145]. Hence, further epidemiological studies specifically designed for elevated liver enzymes and MAFLD are needed in order to better evaluate the effects of PFOA exposure on human liver, as underlined by different authors [135,145]. Of note, some PFAS, such as perfluorooctane sulfonate (PFOS) and perfluorohexane sulfonate (PFHxS), are more likely to cause fatty liver in exposed individuals [148].

In mice, several investigations consistently reported PFOA-induced elevated hepatic triglyceride levels and steatosis [149,150,151,152,153], although this was not confirmed in another study [154]. These discrepancies might be explained by different factors, such as the dose of PFOA and duration of treatment, the mouse strain and the diet. Interestingly, in BALB/c mice, 28 days of PFOA treatment decreased liver triglycerides with the administration of the 0.08 and 20 mg/kg/day doses but increased them with the administration of the 1.25 mg/kg/day dose [150]. PFOA was also shown to induce the accumulation of lipid droplets in hepatocyte nuclei [155]. Because the accumulation of lipid droplets in the nucleus could have significant effects on lipid signaling and nuclear receptor function [156], further investigations are required to determine the exact consequences of this observation. In addition to these in vivo studies, PFOA increased intracellular levels of neutral lipids and triglycerides in different human hepatic cell lines [153,157,158]. However, it should be underlined that these effects were observed for high PFOA concentrations (i.e., 50 μM or more) [153,157,158].

**Figure 3:**
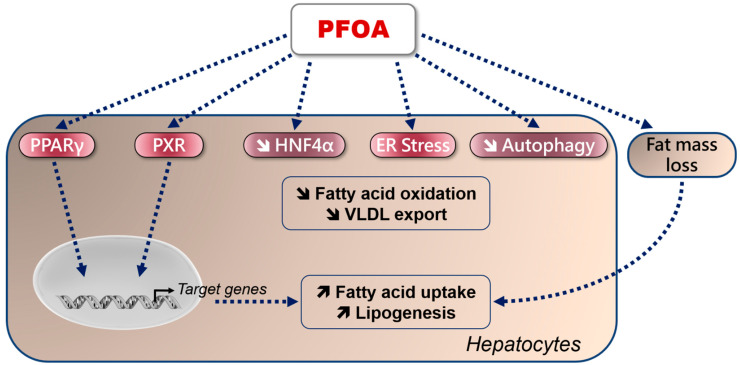
Potential mechanisms involved in PFOA-induced hepatic steatosis. PFAO can directly bind PPARγ or activate PXR, both transcription factors inducing the expression of genes involved in fatty acid uptake and de novo lipogenesis. PFOA reduces the expression and activity of HNF4α, leading to alteration in fatty acid oxidation and very low-density lipoprotein (VLDL) secretion. Reduced VLDL secretion can be secondary to endoplasmic reticulum stress caused by PFOA. Reduction in autophagy by PFOA favors lipid accumulation by reducing the clearance of excessive lipid droplets. Finally, PFOA reduces adiposity, which contributes to an increased lipid flux to the liver in the context of obesity. (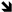: increase; 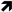: decrease).

From these experimental investigations, several hypotheses can be put forward in order to explain how PFOA induces steatosis (Figure 3), such as the following:

(1) Direct binding to PPARγ [111,159], whose activation increases the expression of *FAT/CD36* and different genes involved in DNL, such as *SREBP1*, acetyl-coenzyme A carboxylase (*ACC*) and *FASN* [90,152]. Of note, SREBP1 can activate PPARγ via the production of fatty acid derivatives acting as endogenous ligand(s) [160]. Hence, it would be interesting to determine whether PFOA can directly activate SREBP1 in a PPARγ-independent manner, which might reinforce DNL stimulation secondary to PFOA-induced activation of PPARγ.
(2) PXR activation. On one hand, previous studies showed that PFOA can activate PXR, in particular the human ortholog [111,161], although this has not been confirmed in other investigations [162,163]. On the other hand, many investigations showed that PXR can trigger a steatogenic response in liver [159,164,165]. Hence, it would be interesting to assess the metabolic effects of PFOA in PXR-knockout mice and in human hepatocytes with PXR silencing.
(3) Reduction in HNF4α protein levels and activity [152,166], an effect which is expected to impair FAO and VLDL secretion [167].
(4) ER stress [157,158], which might also strongly impair VLDL secretion [21,24]. However, ER stress was observed in human hepatic cells with high concentrations of PFOA (i.e., 100 or 200 μM) [157,158].
(5) Impairment of autophagy [153], which might reduce the degradation of excessive lipid droplets [16,20].
(6) Mobilization of lipids from the adipose tissue due to a loss of fat mass [168]. Indeed, some investigations of mice reported that PFOA exposure significantly reduced body weight and adiposity, even when the animals were fed a HFD [154,155,157,168]. PFOA-induced reduction in fat mass in mice might be mainly due to a lower food intake [168,169], possibly via an uncoupling protein 1-dependent mechanism [169].

It is noteworthy that PFOA is a potent activator of PPARα [162,163,170,171]. Although PFOA-induced PPARα activation in rodents can induce hepatic hyperplasia and hepatocarcinogenicity, the use of drugs activating PPARα (e.g., fibrates) in humans have beneficial effects on lipid metabolism, in particular by reducing blood triglycerides and alleviating MAFLD [170,172]. Hence, it is tempting to speculate that PFOA-induced PPARα activation might alleviate its deleterious effects on lipid homeostasis, which might be linked to the activation of other nuclear receptors, such as PPARγ and PXR, as previously mentioned. In keeping with this hypothesis, the PFOA-induced hepatic accumulation of large lipid droplets was observed in male PPARα-null mice but not in male mice expressing human PPARα [171]. In addition to steatosis, PPARα activation might also protect against other liver lesions induced by PFOA, such as bile duct hyperplasia and hematopoietic cell proliferation [173].

Five studies assessed the hepatic effects of PFOA in mice fed with different types of HFDs [154,155,168,171,174]. Unfortunately, two of these studies did not include groups of mice fed a standard diet [171,174]; thus, this does not allow one to determine the additive or synergistic effects between PFOA and high-fat feeding. Nonetheless, in one of the latter studies, PFOA induced significant steatosis in male PPARα-null mice fed a HFD, whereas steatosis was less marked in the non-exposed counterparts [171]. Whether this exacerbation of liver lipid content was associated with hepatic cytolysis remains unknown, as serum ALT and AST activity was not reported [171]. The other study, focused on cholesterol metabolism, reported that PFOA reduced liver cholesterol levels in male and female BALB/c mice fed a HFD but not C57BL/6 mice fed the same diet [174].

The other three investigations of mice reported incomplete or opposing results regarding hepatic lipids and cytolysis between standard diet and HFD [154,155,168]. In one study, PFOA-induced liver triglyceride accumulation was similar between standard-diet- and HFD-fed mice and a synergistic effect between PFOA treatment and HFD was observed for plasma ALT activity [168]. In another study, focused on nuclear lipid droplets, the accumulation of these droplets was similar between standard diet and HFD mice [155]. Unfortunately, this study did not provide information on total liver lipid content and plasma transaminase activity. Lastly, PFOA exposure protected against HFD-induced steatosis, hepatic cytolysis and fibrosis [154]. However, a synergistic effect between early PFOA treatment and HFD was observed regarding PPARα activation and hepatocyte proliferation [154]. In line with this study, recent investigations showed that PFOS protected against HFD-induced hepatic steatosis but serum transaminase activity was not reported [175]. Nonetheless, in this study, PFOS induced steatosis in mice fed a normal diet [175].

In summary, there is no conclusive evidence in mice of any additive or synergistic effect between PFOA exposure and HFD regarding fatty liver, whereas only one study reported a synergistic effect for hepatic cytolysis [168]. Although this study might be in line with some data collected from obese individuals [146,147], further investigations are needed in order to determine the hepatic effects of PFOA in rodent models of obesity. In particular, it would be important to systematically assess both steatosis and hepatic cytolysis, since PFOA might have different effects on these liver lesions [168]. Because PFOA-induced PPARα activation could be a confounding factor in rodent investigations, it would also be of interest to use human cellular models of MAFLD, as recently described for other xenobiotics [39,44,176].

#### 3.2.3. Other Environmental Toxicants

Despite not being described clinically, other environmental toxicants aggravated MAFLD in different rodent models of obesity. For instance, this was reported for diesel exhaust particles [177], hexabromocyclododecane (HBCD) [178], perchloroethylene (PCE) [179], 2,3,7,8-tetrachlorodibenzo-*p*-dioxin (TCDD) [180], nonylphenol [181] and tetrabromodiphenyl ether (BDE-47) [182]. Aggravation of fatty liver by TCDD and BDE-47 might be linked with aryl hydrocarbon receptor (AhR) activation and the subsequent increased expression of *FAT/CD36* [180,182], whereas HBCD and nonylphenol might act via PPARγ activation [178,181]. Regarding perchloroethylene, which aggravated both fatty liver and hepatic cytolysis (as assessed by serum ALT activity) in HFD-fed mice, the precise mechanism of MAFLD worsening was unfortunately not delineated [179]. Nevertheless, this study showed that MAFLD was associated with the hepatic accumulation of perchloroethylene and its major oxidative metabolite, trichloroacetate [179]. Hence, the worsening of MAFLD induced by perchloroethylene might be, at least in part, secondary to an increase in its bioavailability. However, the underlying mechanisms whereby these molecules aggravate MAFLD are poorly characterized and warrant further investigations.

### 3.3. Ethanol

Even though ethanol can be found in the blood of non-alcoholic individuals [183], this molecule is mentioned in this review because its overconsumption is a major issue for public health. Regarding the liver, sustained high alcohol consumption (>20 and 30 g/day of alcohol for women and men, respectively) leads almost always to hepatic diseases such as acute hepatitis, fatty liver, steatohepatitis, cirrhosis and hepatocellular carcinoma [184,185]. Indeed, alcohol intoxication induces several deleterious effects in hepatocytes and other liver cells, such as mitochondrial dysfunction, increased lipogenesis, CYP2E1 induction with subsequent ROS overproduction and oxidative stress, impairment of autophagy, ER stress and increased production of proinflammatory and profibrotic cytokines. Readers are invited to peruse recent reviews for further information regarding the mechanisms of ethanol-induced hepatic toxicity [184,185,186,187,188,189,190,191].

There is now strong evidence that alcohol abuse and obesity (or metabolic syndrome) synergistically increase the risk and severity of different hepatic diseases, including fatty liver, steatohepatitis, cirrhosis and hepatocellular carcinoma [192,193,194,195]. Experimental investigations attempted to determine how ethanol and obesity synergistically increase hepatic toxicity in particular regarding steatosis, hepatocyte cell death and inflammation. Unsurprisingly, different mechanisms have been proposed, including impaired mitochondrial function and biogenesis [196,197], reduced expression of PPARα [196,198] or AMP-activated protein kinase (AMPK) [199], induction of CYP2E1 and oxidative stress [200,201], unfolded protein response and ER stress [196,202], increased expression of tumor necrosis factor alpha (TNF-α) [203] or Fas ligand [204,205] and activation of different proinflammatory pathways [206,207,208,209]. Interestingly, ethanol exerts different deleterious effects on WAT such as lipoatrophy, increased lipolysis and enhanced secretion of proinflammatory adipokines [210,211,212], which might also explain why alcohol intoxication is particularly hepatotoxic in obese individuals [195].

Finally, recent investigations of different experimental models of MAFLD suggested that benzo[a]pyrene exposure might even further aggravate the progression of steatosis to steatohepatitis induced by ethanol [176,213,214,215]. Potential mechanisms involved in such progression include overproduction of ROS and nitric oxide [176,214], mitochondrial dysfunction [176], increased expression of proinflammatory cytokines [213] and plasma membrane remodeling [215].

## 4. Key Mechanisms Involved in Xenobiotic-Induced Aggravation of MAFLD

Collectively, the aforementioned studies clearly indicate that xenobiotics can worsen MAFLD via different mechanisms, as illustrated in Figure 1 and Figure 2. The exacerbation of pre-existing fatty liver seems to frequently involve increased DNL, either directly via the activation of different lipogenic transcription factors such as PPARγ and PXR, or indirectly through hyperinsulinemia or hyperglycemia, which can, in turn, activate SREBP1c and ChREBP, respectively (Figure 1). Furthermore, xenobiotics might worsen steatosis by impairing VLDL secretion, in particular as a consequence of ER stress. The impairment of mitochondrial FAO might also be involved with some chemicals, in particular with drugs and ethanol, whose intrahepatic concentrations can be relatively high. Notably, xenobiotic-induced impairment of VLDL secretion and mitochondrial FAO is expected to curb two major metabolic adaptions occurring during MAFLD, as mentioned previously. Xenobiotic-induced worsening of pre-existing necroinflammation and fibrosis could involve chronic ROS overproduction and subsequent oxidative stress, which can favor the production of pro-inflammatory cytokines (e.g., TNF-α and interleukin-1β) and transforming growth factor-β (TGF-β), a key pro-fibrotic cytokine (Figure 2). In the setting of MAFLD and xenobiotic exposure, ROS overproduction seems to be mainly secondary to mitochondrial dysfunction, whereas the induction of CYP2E1 can play a role with some compounds such as ethanol.

## 5. Conclusions

MAFLD may be worsened by different drugs and environmental toxicants but also by ethanol, which is consumed by millions of people worldwide. However, except for a handful of xenobiotics (Table 1), there is currently a blatant lack of information regarding the whole spectrum of molecules able to aggravate MAFLD. Accordingly, there is an urgent need to determine which compounds could pose a specific risk for overweight or obese individuals. To this end, experimental investigations using cellular models of MAFLD might allow a great number of xenobiotics to be screened [47,176,216]. Although there is increasing evidence that many xenobiotics might alter the insulin signaling pathway [217,218,219,220], it should be pointed out that these in vitro models cannot detect MAFLD worsening due to extra-hepatic effects such as insulin resistance (Figure 1).

It would also be important to determine if the mechanisms of xenobiotic-induced MAFLD worsening in obese patients are the same as those triggering steatosis and steatohepatitis in lean individuals. To this end, experimental investigations of animal and cellular models could greatly help to answer this question. Indeed, the worsening of fatty liver in obese animals (or steatosis in cellular models of MAFLD) associated with the lack of hepatic lipid accumulation in lean animals (or in non-steatotic hepatic cells) would decipher specific mechanism(s) in the context of obesity and MAFLD. These investigations might also unveil the existence of additive or synergistic effects between the investigated compounds and MAFLD regarding the aggravation of liver disease.

Finally, investigators should seek out the possible toxicological interactions between pharmaceuticals, environmental pollutants and even alcohol. This is a major issue because obese people are often polymedicated and are expected to be exposed to some environmental contaminants and to consume different types of alcoholic beverages. In these individuals, different factors might trigger fatty liver or favor its progression to steatohepatitis and cirrhosis. Thus, we propose to coin the term “mixed-origin fatty liver disease” (MOFLD), which could better reflect the multiple causes leading to fatty liver disease, such as overnutrition, drugs, environmental toxicants and excessive alcohol consumption. It should finally be underlined that, in obesity, tissues other than the liver can be particularly injured by xenobiotics, such as heart [221,222,223] and kidneys [224,225,226]. Accordingly, xenobiotic-induced toxicity in obese individuals should concern hepatologists but also physicians from other medical specialties.

## Figures and Tables

**Table 1:** Drugs and environmental toxicants shown or suspected to worsen obesity-related fatty liver disease ^1^.

Drugs	Environmental Contaminants
**Corticosteroids (Corticosterone, Dexamethasone)**	**Bisphenol A (BPA)**
Irinotecan ^2^	Diesel exhaust particles
Methotrexate ^2^	Hexabromocyclododecane (HBCD)
Nucleoside reverse transcriptase inhibitors (didanosine, stavudine) ^2^	Nonylphenol
Pentoxifylline ^2^	Perchloroethylene
Phenobarbital ^2^	**Perfluorooctanoic acid (PFOA)**
Tamoxifen ^2^	Tetrabromodiphenyl ether (BDE-47)
Tetracycline ^2^	2,3,7,8-Tetrachlorodibenzo-*p*-dioxin (TCDD)
**Thiazolidinediones (rosiglitazone, troglitazone, pioglitazone)**	

^1^ See text for details. ^2^ Refer to previous reviews [40,47] for more information. Compounds in bold letters are discussed in detail in the manuscript, in particular regarding the involved mechanism(s). Ethanol is not mentioned in this table because this molecule is not typically considered as a xenobiotic.
